# The Diagnostic Accuracy of the M2 Pyruvate Kinase Quick Stool Test- A Rapid Office Based Assay Test for the Detection of Colorectal Cancer

**DOI:** 10.1371/journal.pone.0131616

**Published:** 2015-07-09

**Authors:** Suresh Sithambaram, Ida Hilmi, Khean-Lee Goh

**Affiliations:** Division of Gastroenterology and Hepatology, Department of Medicine, University of Malaya, Kuala Lumpur, Malaysia; University Medical Center of Princeton/Rutgers Robert Wood Johnson Medical School, UNITED STATES

## Abstract

**Background:**

M2 pyruvate kinase (M2PK) is an oncoprotein secreted by colorectal cancers in stools. This the first report on the accuracy of a rapid stool test in the detection of colorectal cancer (CRC).

**Objective:**

To determine the sensitivity, specificity and positive and negative predictive value of a rapid, point of care stool test M2 PK- the M2PK Quick.

**Methods:**

Consecutive cases of endoscopically diagnosed and histological proven CRC were recruited. Stools were collected by patients and tested with the immunochromatographic M2PK Quick Test (Schebo Biotech AC, Giessen, Germany). Controls were consecutively chosen from patients without any significant colorectal or gastrointestinal disease undergoing colonoscopy. CRC was staged according to the AJCC staging manual (7^th^ Edition) and location of tumor defined as proximal or distal.

**Results:**

The sensitivity, specificity, positive predictive value, negative predictive value and overall accuracy were: 93%, 97.5%, 94.9%, 96.5% and 96.0% respectively. The positive predictive value for proximal tumors was significantly lower compared to distal tumors. No differences were seen between the different stages of the tumor.

**Conclusions:**

The M2-PK Quick, rapid, point-of-care test is a highly accurate test in the detection of CRC. It is easy and convenient to perform and a useful diagnostic test for the detection of CRC in a clinical practice setting.

## Introduction

Stool tests that have been widely used in the detectionand screening of colorectal cancer (CRC) are based on the testing for occult blood in stools. The guaic based tests have been in use for alongtime but suffers from the drawback of a high false positive resultdue to the inherent nature of the test which depends on the oxidative capacity of the guaicsubstrate. In general the guaic based fecal occult tests have limitedsensitivity and specificity. In one study,the sensitivity of guaiac based test (HemoccultSensa) was 79.4% and the specificity was 86.7% [1]. The guiac test is inconvenient to perform as patients have to go on a restricted diet several days before the test which includes avoiding various types of food that may cause false peroxidase reaction or are antioxidants and NSAIDs and aspirin. These problems have been overcome by the newer fecal Immunochemical test (FIT)which detects the globin moiety of humanhemoglobin. Globin which arises from upper GI tract is rapidly degraded by digestive enzymes. Therefore these test arehighly selective for occult bleeding of colorectal in origin. The sensitivity of FIT for detection of CRC is about 70–90%[2–6].

M2-pyruvate kinase (M2-PK) is an isoenzyme of pyruvate kinase, which is a key enzyme in glycolysis where it catalyzes the conversion of phosphoenolpyruvate to pyruvate. This isoenzyme is generally a highly active, tetrameric form. However in tumor tissue, on exposure to oncoproteins, M2-PK is converted into a less active dimeric form, a change which is necessary for tumor metabolism [7]. M2-PK is not specific for any tumour and has been reported to be found in various cancers including kidney, esophageal, gastric, pancreatic, colorectal, lung, ovarian and breast cancer [8].M2-PK has been found in blood and stool in various cancers and thus been used in cancer screening or detection and follow-up to detect recurrence.

In CRC,tumor M2-PK is shed in the colonic lumen andtherefore can be detected in the stools of patients. This forms the basis of the M2-PK stool test. The use of M2-PK test stool was first reported by Hardt et al in 2003 whenthe authors reported quantification of M2 PK levels in stools and suggested that it may be useful in CRC screening [9]Since then, several validation studies have now been performed; mainly on western patients and shown to have a fairly high sensitivity and specificity.

Recently a newer version of the M2-PK test- the rapid office based qualitative test–M2PK Quick was introduced for clinical use. This is the first study to validate the M2PK Quick for the detection of CRC.The aim of this study is to determine the diagnostic accuracy of the M2-PK Quick stool test in the detection of CRC.

## Methods

This is a case control study that was carried out at the University of Malaya Medical Centre (UMMC) fromJanuary 2013 to December 2013. Approval for the study was specifically obtained from the Institutional Review Board of the hospital: the ethics committee of the UMMC and the study was carried out in accordance to ICH-GCP guidelines. A patient information leaflet and a written consent form, which is standard policy, were approved by the committee. The study aims and procedures were explained in detailed to all participants. The patient information leaflet and written consent were given to all participants. All written consent forms obtained were kept in the case record files of each patient.

Consecutive patients with CRC histologically confirmed on colonoscopy and biopsy were recruited for the study. The casesconsisted of patients undergoing CRC screening(asymptomatic) or patients who presented with symptoms and signs suggestive of CRC (tenesmus, alteration in bowel habit, PR bleed and weight loss). Controls consisted of consecutive patients who had undergone screening colonoscopy with no evidence of CRC, adenomasnor dysplasia and wereotherwise healthy. Control subjects who had any clinical suspicion of any GI tumor were excluded.The recruitment ratio of cases and controls was 1: 2. The diagnosis of CRC was confirmed on biopsies and resected surgical samples sent for histopathologicalexamination.CRC patients were subcategorized according to tumor location and staging. For staging,the American Joint Committee on Cancer (AJCC)classification based on CT scan and on operative findings was used. Tumor location was categorized as either proximal (cecum,ascendingcolon,hepaticflexure,transverse colon and splenic flexure) or distal (descending colon,sigmoidcolon,rectum and anal canal).

### Collection of stool specimen and testing for M2-PK

The stool testing for M2PK was carried out by a single trained personnel who was blinded to the results of the colonoscopy.Stools were collected either before the colonoscopy and bowel preparation was carried out or at least one week after the colonoscopy. “Fresh” stool samples were tested as soon as collected. If testing were not possible, stool samples were kept at 4^0^ C in the refrigerator for no longer than 24 hours.

Testing for the M2-PK protein in the stools of patients was carried out by using the rapid, point of care,M2-PKQuickTest(ScheBoBiotech AG, Giessen, Germany).Thetest was carried out according to the manufacturer’s instructions. The interpretation based on the appearance of bands in the test (T) and control (C) areas of the test strip when a solubilized stool sample is placed. The M2PK Quick test is an immunochromatographic rapid test. When the M2PK protein is present in stools, it reacts with a monoclonal antibody bound to gold particles and forms a gold-labelled complex, called the antibody-M2PK complex. This complex is then migrates along the membrane and reached the Test line (T) which has second monoclonal antibody will bind to this antibody-M2PK complex, and subsequently develop a pink colour line at T.

### STARD Guidelines

The study was reported according to Standards for the Reporting of Diagnostic accuracy studies guidelines.

### Statistical analysis

Statistical analysis was done by using SPSS version 16 for Windows (SPSS Inc, Chicago, Illinois). For the baseline demography, Student t test and Chi-square testing were used where applicable. The sensitivity, specificity and positive and negative predictive values of the rapid serology test were calculated against the gold standard for diagnosis of CRC based on histological confirmation. 95% confidence intervals (C.I.) were calculated for proportions of these values.

## Results

**Fig 1:**
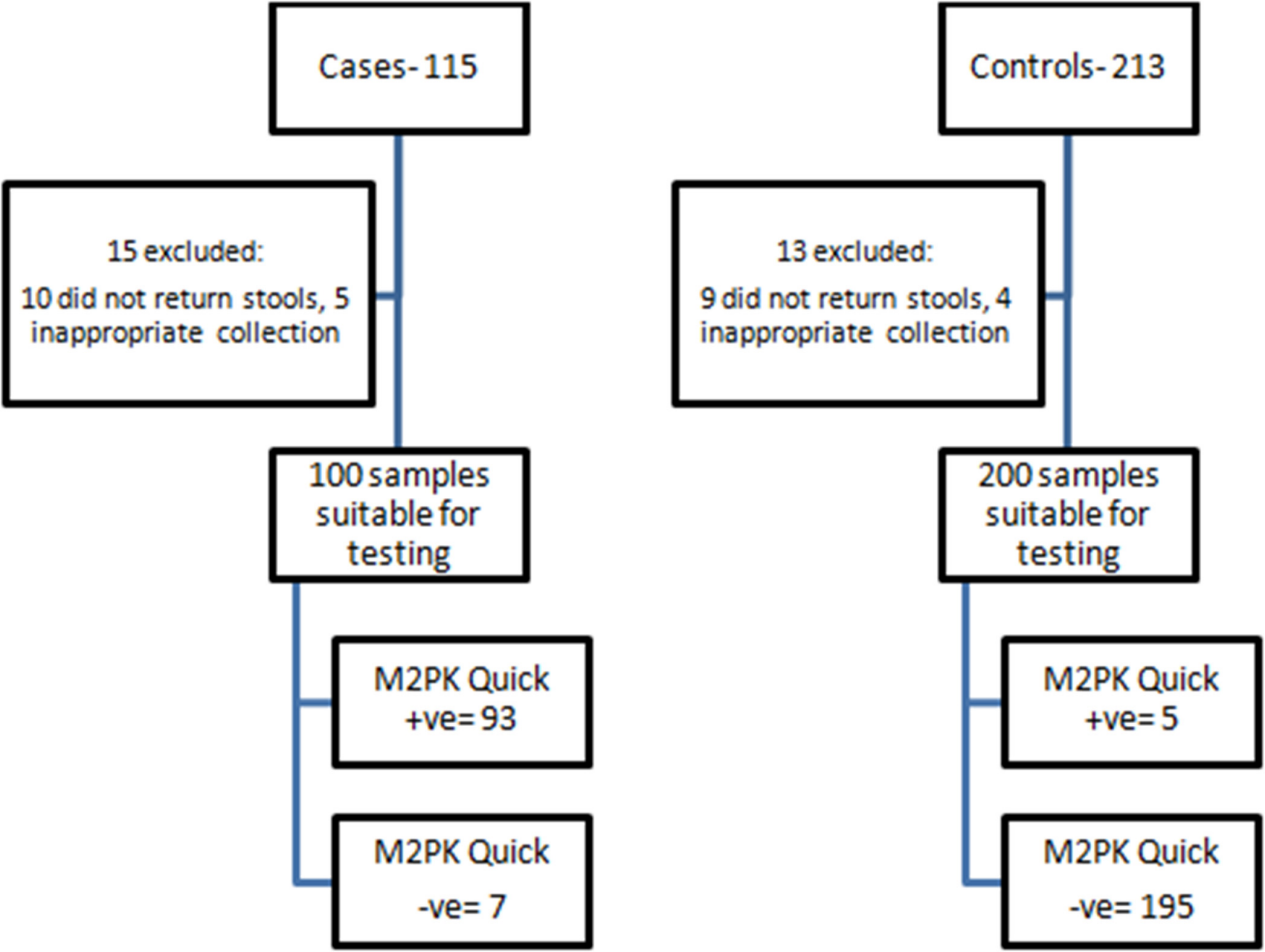
Flow Diagram of Subject recruitment and Diagnostic testing- CRC cases and Controls.

One hundred and fifteen patients were diagnosed to have CRCduring the period of the study. However stool samplescollected appropriately were obtained from only 100 patients and tested for the M2-PK protein. Likewise,stool samples were requested from 213 subjects with normal colonoscopy but only 200 cases were appropriately collected and tested for the M2-PK protein (Fig 1).

The baseline demography of the cancer and control cases are summarized in Table 1.There were more Chinese in the cancer group compared to the other two main ethnic races in Malaysia, the Malays and the Indians. This is in keeping with data from the Malaysian National Cancer Registry which shows the highest incidence of CRC is among the Chinese ethnic group. There were no significant differences in the mean age and gender distribution among the cases and the controls.

**Table 1:** Basic demography of patients and controls.

	Cancer	Controls	P value
**Mean age ± S.D.**	65.31±13.16	60.31 ± 11.78	*p* = 0.112
**Gender, n (%)**			*p* = 0.191
**Male**	58 (58%)	100 (50%)	
**Female**	42(42%)	100 (50%)	
**Ethnicity**			*p* <0.05
**Malay**	25 (25%)	100(50%)	
**Chinese**	62 (62%)	60(30%)	
**Indian**	13 (13%)	40(20%)	

The sensitivity, specificity, positive predictive value, negative predictive value and overall accuracy of the M2-PK test were as follows; 93%, 97.5%, 94.9%,96.5% and 96.0% respectively (Table 2). In the 5 patients with a false positive M2-PK test, a gastroscopy, a repeat colonoscopy and basic laboratory tests were carried out which were all found to be negative.

**Table 2:** Overall sensitivity, specificity, positive predictive value (PPV), negative predictive value (NPV) and diagnostic accuracy of the M2PK Quick test.

	M2PK positive	M2PK negative	Sensitivity % (95% CI)	Specificity % (95% CI)	PPV % (95% CI)	NPV % (95% CI)	Accuracy % (95% CI)
**Cancer**	93	7	93 (86.3–96.6)	97.5 (94.3–98.9)	94.9 (88.6–97.8)	96.5 (93.0–98.3)	96.0 (93.1–97.7)
**Controls**	5	195					

### Sub-analysis based on tumor location and staging

The diagnostic accuracy of the tests according to tumor location. Out of the 100 patients, 87 were distal tumors and 13 were proximal. The sensitivity, specificity, positive predictive value and negative predictive value of the M2-PK test according to tumor location is summarized in Table 3. There were nosignificant differences in terms of the specificity, negative predictive value and overall accuracy of the test. Thesensitivity was numerically lower but only the positive predictive value was significantly lower for proximal compared to distal tumors (p = 0.010).

**Table 3:** Sensitivity, specificity, positive predictive value (PPV), negative predictive value (NPV) and diagnostic accuracy of the M2PK Quick test according to tumor location.

	M2PK positive	M2PK negative	Sensitivity, %(95% CI)	Specificity, %(95% CI)	PPV, %(95% CI)	NPV, %(95% CI)	Accuracy, %(95% CI)
**Proximal cancer**	12	2	85.7(60.1–96.0)	97.5(94.3–98.9)	70.6(46.9–86.7)	99.0(96.4–99.7)	96.7(93.4–98.4)
**Distal cancer**	81	5	94.2(87.1–97.5)	97.5(94.3–98.9)	94.2(87.1–97.5)	97.5(94.3–98.9)	96.2(93.7–98.1)
**Control**	5	195					

The sensitivity, specificity, positive predictive value and negative predictive value of the M2-PK test according to tumor stagingis summarized in Table 4. There were nosignificant differences in terms of the sensitivity, specificity, positive and negative predictive values and accuracy of the M2-PK test for the different tumor stages.

**Table 4:** Sensitivity, specificity, positive predictive value (PPV), negative predictive value (NPV) and diagnostic accuracy of the M2PK Quick test according to tumor staging.

AJCC Stage	M2PK positive	M2PK negative	Sensitivity, %(95% CI)	Specificity, %(95% CI)	PPV, %(95% CI)	NPV, %(95% CI)
**Stage 1**	16	1	94.1(73.0–99.0)	97.5(94.3–98.9)	76.2(54.9–89.4)	99.5(97.2–99.9)
**Stage 2**	16	2	88.9(67.2–96.9)	97.5 (94.3–98.9)	76.2(54.9–89.4)	99.0(96.4–99.7)
**Stage 3**	27	1	96.4(82.2–99.4)	97.5(94.3–98.9)	84.4(68.3–93.1)	99.5(97.2–99.9)
**Stage 4**	28	3	90.3(75.1–96.7)	97.5(94.3–98.9)	84.8(69.1–93.4)	98.5(95.6–99.5)
**Control**	5	195				

## Discussion

The M2-PK fecal test is a novel test in the detection of CRC. As proliferatingtumour cells shed M2-PK readily from the surface of the tumor, this test would allow us to reliably detect cancers from the gastrointestinal tract.

Two types of fecal M2-PK test are available: A quantitative test, ELISAbased laboratory test and aqualitativepoint of care immunochromatographic test. An office-based, point of care test for stool detection is not as practical as a similar test for blood samples as result as will not be immediately available. Stools will usually have to be collected at a separate occasion and usually at home. However,this test allows the busy doctor in clinical practice to perform the test in his own clinic with results being available almost as soon as the stool sample is submitted.On other hand, a quantitative test requires the stool sampleto be sent to a well fairly equipped laboratory with an ELISA reader and withtrained personnel, requiring several hours before results can be obtained.

The M2PK quantitative test has been widely tested and have shown a sensitivity ranging from 73–97% and specificity from 78.6–100.0% [10–17].(Table 5). However, to date there have been no validation of the rapid point of care M2-PK Quick stool test and our study isthefirst reported study. In our experience, wehave found the rapid M2-PK fecal test to be a highly accurate test with an overall diagnostic accuracy of 96% with a sensitivity of 93% and a specificity of 97.5%.We found that the test is easy and convenient to perform with minimal handling of stools. The test can be performed quite rapidly (within 10min) and the results based on colorimetric changes with the appearance of vertical bands, easy to read.

**Table 5:** Sensitivity and Specificity of Fecal M2-PK tests and gFOBT and iFOBT- published results.

	Fecal Test	Sensitivity, %	Specificity, %
Hardt^11^	M2-PK	73.3	77.8
Naumann^10^	M2-PK	85.2	65.3
	gFOBT	63	86.7
Vogel^12^	M2-PK	77.3	71.8
	gFOBT	27.3	89.1
	iFOBT	90.9	93.8
Tonus^14^	M2-PK	77.8	92.9
Shastri^13^	M2-PK	81.1	71.1
	gFOBT	36.5	92.2
Koss^16^	M2-PK	90.6	92.3
Mulder^15^	M2-PK	84.6	90.5
	iFOBT	92.3	96.8
Shastri^17^	M2-PK	78.2	73.8
	iFOBT	70.9	96.3
This study	M2-PK (Quick Test)*	93	97.5

*Case-control validation study

Our study has also shown that the test is highly accurate regardless of the stage of the tumor although this is in contrast to other studies. For example,Shastri and colleagues found a significantly higher proportion of patients with Dukes C and D cancers (89.5%) vs. Dukes A and B cancers (63.9%) having a positive quantitative M2-PK test [17]. This is based on a predefined cut-off level of 4 u/ml which is the same cut off value used in our rapid qualitative test. In our study, it also appears that the test is highly accurate regardless of tumor location although the sensitivity appears to be slightly lower in proximal compared to distal tumors.However, the numbers for each tumor stage in our study are small with wide confidence intervals, and the results must therefore be interpreted with caution.

How has the M2PK test performed in the detection of CRC? In our case control study we have included patients with overt CRC and did not have patients withvery early CRC confined to the mucosa. Cases with colorectal adenomas were not included in the study. In a fairly large screening study in a German population, using the laboratory based EISA assay and looking specifically at colorectal adenomas, stool M2-PK showed a low sensitivity of only 22% and 23% for advanced and non-advancedadenomas(early and intermediate)respectively although a specificity of 82% was reported [18]. Similarly in a Dutch study by Mulder et al, with a smaller sample size, the authors found the sensitivity of 27% and 29% for advanced and non-advanced adenomasrespectively with a specificity of 90% [15]. In another German study by Shastri et al, the sensitivity of detecting large adenomas(defined as greater than 1cm)was 44.4% for proximal lesions and 50.0% for distal lesions [17].Therefore, M2-PK appears to have limited role in detecting non advanced and advanced adenomas.

The other point to consider was that both symptomatic and asymptomatic patients were recruited into the study who were consecutively found to have CRC. It is not clear whether or not the M2-PK test will have similar diagnostic accuracy in a purely asymptomatic population as part of a CRC screening program as one could argue that symptomatic patients require a colonoscopy anyway. However, in a developing country such as Malaysia where endoscopic services remain limited, the M2-PK test may allow risk stratification in terms of need and timing for colonoscopy.

Another confounding factor in clinical practice would be the presence of concomitant inflammation in the colon. Positive tests have also been reported in inflammatory bowel disease which constitutes a “false positive” test. In a small group of patients, Mulder etal showed that in IBD patients, the M2-PK test was positive in 15 of 19 (78.9%) [15]. In a study by Walkoniak and colleagues, the level of M2-PK was quite significantly higher in patient with pouchitis following ileo-anal anastomosis for ulcerative colitis [19]. In another multicenter study, up to 88% of patients with ulcerative colitis and Crohn’s Disease had elevated M2-PK levels [20]. In a population where IBD is not uncommon, these findings may negate against the use of this test for detection of CRC. In a study from neighboring Indonesia, the authors had reported the value of M2-PK positive tests in detecting cancers in patient with IBD, infective colitis and amebic colitis [21]. However, there was no mention of the M2-PK level or the number of “false positive” tests in these patients in the absence of cancer.A prospective study studying the accuracy of this test across the whole spectrum of colonic disease in clinical practice will be important and will allow us to better define its role in the screening of CRCs.Finally, the presence of stool M2-PK does not necessarily indicate the presence of CRC alone and care should be taken to exclude disease in the upper gastrointestinal tract and in the pancreatico-biliary tree.

The most widely used stool test for screening for CRC remain the fecal occult based tests and there have been studies comparing the accuracy of M2-PK compared to these tests. When compared to guaic based FOBT for the detection of CR adenomas, a meta-analysis by Tonus et al amalgamating the results of 4 studies showed a significant better detection rate with the M2PK test for adenomas both less and morethan1cm in size [22]. The quantitative M2-PK test has been compared to the newer immunochemical fecal occult blood testing. Shastri et al showed that the sensitivity of both the iFOBT and the M2-PK test were very similar for early stage as well as advanced CRC [17]. In another study, M2-PK stool test was compared against two iFOBT tests for detection of adenomas and showed again equivalent diagnostic accuracy with all 3 tests showing a low sensitivity of 28–40% [15].Therefore, the M2-PK test is a viable alternative to currently available fecal occult blood tests.

However as the main utility of stool based testing is for screening in an asymptomatic population, a future study should be planned to look at the sensitivity, specificity and overall accuracy of the rapid point ofcare M2-PK test in a screening population and compare it to the rapid point of care (qualitative) FIT. Another possibility would be the use of both tests in combination although the cost effectiveness of this strategy needs to be determined. A study by Leen at al [23] found that the addition of the quantitative M2-PK test to the standard FIT detected additional adenomas at an earlier stage

## Conclusions

The M2-PKQuick, rapidpoint-of-care test which has been shown to be a highly accurate test in the detection of CRC. It is easy and convenient to perform in a doctor’s office. It is certainly a useful diagnostic test for the detection of CRCin a clinical practice setting.

## Supporting Information

S1 Checklist STARD checklist for reporting of studies of diagnostic accuracy (DOCX)
